# Comprehensive Analysis of *CaFAD* Genes Involved in Fatty Acid Accumulation in *Coffea arabica* and Functional Characterization of *CaFAD8* in Transgenic *Arabidopsis thaliana*

**DOI:** 10.3390/ijms26031023

**Published:** 2025-01-25

**Authors:** Zhenwei Zhang, Xuejun Li, Meijun Qi, Sumera Anwar, Butian Wang, Yu Ge

**Affiliations:** 1 College of Tropical Crops, Yunnan Agricultural University, Pu’er 665099, China; zhangzhenwei202309@163.com (Z.Z.); 2003056@ynau.edu.cn (X.L.); qimeijun2003@163.com (M.Q.); wangbutian@stu.ynau.edu.cn (B.W.); 2 Yunnan Provincial Key Laboratory of Coffee, Yunnan Agricultural University, Pu’er 665099, China; 3 Department of Botany, Government College Women University Faisalabad, Faisalabad 38000, Pakistan; anwer_sumera@yahoo.com

**Keywords:** coffee plant, *CaFAD* gene family, subcellular localization, expression pattern, gene function identification

## Abstract

The quality of *Coffee arabica* L. beans, particularly the aroma, is a key determinant of commercial value. Fatty acids, as precursors of volatile aroma compounds, play a crucial role in this quality. Screening and identification of their related genes are of particular significance. This study identified 21 members of the *CaFAD* gene family in the *C. arabica* genome using bioinformatics tools. Gene duplication events observed in the *CaFAD* gene family were likely driven by natural selection and mutation pressure, with natural selection being more prominent. Transcriptome sequencing, qRT-PCR, and fatty acid profiling across four fruit developmental stages revealed that *CaFAD8* was closely associated with fatty acid synthesis regulation. Fatty acid content was initially high but decreased during the later stages, while *CaFAD8* expression showed an inverse pattern. Subcellular localization indicated that *CaFAD8* functions primarily on the inner membrane. *CaFAD8-OE* heterologous expression experiment in *Arabidopsis thaliana* reduced the total fatty acid content in seeds but increased unsaturated fatty acids, including oleic, linoleic, and linolenic acids. These findings suggest that *CaFAD8* promotes fatty acid unsaturation and provides insights into fatty acid metabolism in *C. arabica.* This study offers a foundation for understanding *CaFAD* gene regulation and supports breeding strategies for high-oil *C. arabica* varieties.

## 1. Introduction

The coffee plant is a perennial evergreen shrub or small tree belonging to the genus Coffea in the Rubiaceae family. Due to its high economic and nutritional value, it is widely cultivated in tropical and subtropical regions, particularly in developing countries [1,2]. Among approximately 124 species in the genus Coffea, *Coffee arabica* L. (Arabica coffee) and *Coffee canephora* Pierre ex A. Froehner (Robusta coffee) dominate global coffee production, accounting for 60% and 40%, respectively [3].

The flavor and aroma produced during the roasting process of coffee beans depend largely on their chemical components, including proteins, amino acids, sugars, and fatty acids [4,5,6]. Fatty acids, in particular, serve as essential precursors for the formation of volatile compounds during roasting, impacting aroma and flavor [7,8]. In *C. arabica* seeds, fatty acid typically ranges from 82 to 204 g/kg, with palmitic acid, stearic acid, oleic acid, linoleic acid, and arachidic acid as major components [9]. Among these, palmitic acid, linoleic acid, and oleic acid dominate, often accounting for over 50% of the total fatty acid content [10,11,12,13]. A study on coffee beans from eight countries found that linoleic acid had the highest proportion, accounting for more than 40% of the total fatty acids, followed by palmitic acid with a proportion of more than 30%.

In plants, fatty acids are classified into saturated fatty acids and unsaturated fatty acids, with polyunsaturated fatty acids being dominant. Their synthesis involves complex metabolic pathways catalyzed by desaturases [14]. These desaturases can be further subdivided into two major categories: soluble proteases and membrane-bound proteases. The five major subfamilies of desaturases are acyl-CoA-desaturase (ADS), sphingolipid Δ-4 desaturase (DES), Δ-9 stearoyl-ACP desaturase (SAD), acyl-lipid desaturase (FAD), and sphingolipid Δ-8 desaturase (SLD) [15].

Stearoyl-ACP desaturase (SAD) is the only soluble desaturase, and its role is to introduce a double bond at the ∆9 position of stearic acid (C18:0) before the formation of the glycerides of fatty acids, thereby forming monounsaturated oleic acid (C18:1) [16]. FAD2 is a membrane-bound desaturase, mainly distributed on the endoplasmic reticulum and chloroplasts, and can be further divided into omega-3 (ω-3) desaturase (FAD3/FAD7/FAD8) and omega-6 (ω-6) desaturase (FAD2/FAD6 and FAD4). ω-3, ω-6, and FAD4 are involved in the synthesis pathways of polyunsaturated fatty acids such as linoleic acid (18:2), linolenic acid (18:3), and palmitoleic acid (16:1), respectively [17]. FAD2 is located downstream in the oleic acid synthesis pathway, and its main function is to regulate the conversion of oleic acid to linoleic acid. Therefore, FAD2 is a key enzyme that determines the contents of oleic acid and linoleic acid in plant seeds [18].

Despite their importance in determining coffee quality, the molecular mechanisms underlying their synthesis and regulation in *Coffea arabica* remain poorly understood. Identifying and characterizing genes involved in fatty acid biosynthesis could enhance breeding strategies for high-quality, high-oil coffee varieties. The *FAD* gene family is known to play a crucial role in fatty acid desaturation and accumulation in plants. However, the specific members of the *CaFAD* gene family and their contributions to fatty acid synthesis in *C. arabica* have not been systematically studied.

This study hypothesizes that specific members of the *CaFAD* gene family, identified through bioinformatics and expression analysis, regulate fatty acid biosynthesis in *C. arabica* seeds by influencing unsaturation levels. Functional validation of candidate genes in a heterologous *Arabidopsis thaliana* system will provide insights into their roles in lipid metabolism and their potential contribution to aroma precursor formation.

This study aims to (a) identify and characterize *CaFAD* gene family members involved in fatty acid biosynthesis in *C. arabica*, (b) analyze the unsaturated fatty acid content and expression patterns of *CaFAD* genes during seed development, and (c) functionally validate *CaFAD8* through heterologous expression in Arabidopsis thaliana.

## 2. Results

### 2.1. Identification and Physiochemical Analysis of CaFAD Gene Family Protein in C. arabica

A total of 21 candidate *CaFAD* gene family members (Table S1) were identified in *Coffea arabica* L. The number of amino acids in the proteins of these members ranged from 329 to 448, with molecular weight varying between 38,427.47 and 51,654.48 kDa. The isoelectric points (pi) spanned from 6.04 to 9.71, indicating a wide range of acidic and basic protein characteristics. Notably, five members exhibited pi values below 7. While the majority displayed pi values above 7, indicating the dominance of basic amino acids within the family.

The instability index ranges from 28.27 to 55.69. The instability index of 9 members was less than 40 and was predicted to be stable, with CaFAD4 being the most stable (28.77). At the same time, that of 12 members was greater than or equal to 40 and classified as unstable, with CaADS2 (55.69) being the most unstable.

The grand average of hydropathicity (GRAVY) values for most proteins was negative, indicating that the majority of CaFAD members are hydrophobic. Only two proteins, CaFAD2 and CaFAD5, exhibited slightly positive GRAVY values (0.005 and 0.015, respectively), suggesting weak hydrophobicity.

### 2.2. Phylogenetic Tree Analysis of the CaFAD Gene Family in C. arabica

**Figure 1:**
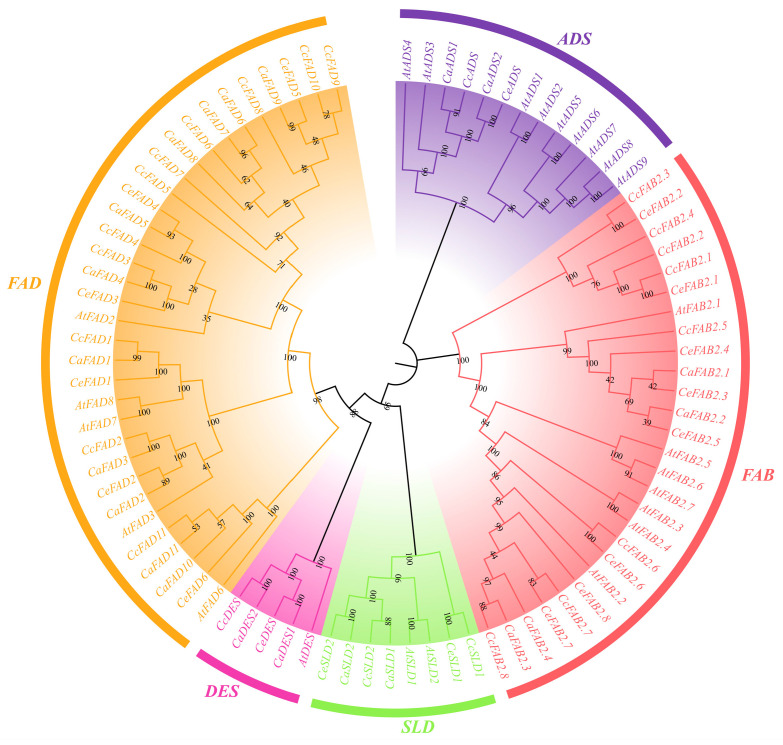
Phylogenetic tree of the *CaFAD* family of *C. arabica* and their homologs in *A. thaliana*, *C. eugenioides*, and *C. canephora*.

A phylogenetic tree (Figure 1) was constructed using the homologous fatty acid desaturases protein sequences from four species: *C. arabica*, *C. canephora*, *C. eugenioides*, and *A. thaliana*. Based on the sequence homology and evolutionary distances, the *FAD* gene family was grouped into five distinct subfamilies: acyl-CoA desaturases (ADSs), fatty acid biosynthesis (FAB), sphingolipid desaturases (SLDs), sphingolipid Δ-4 and Δ-8 desaturases (DESs), and fatty acid desaturases (FADs). Among them, the FADs, the largest and most diverse subgroup, are further divided into FAD2, FAD3/7/8, and FAD6. CaFAD8 clustered closely with AtFAD8 (*A. thaliana*) and genes from *C. canephora* and *C. eugenioedes*, indicating its functional conservation in plastidial ω-3 desaturation pathways.

### 2.3. Analysis of Conserved Motifs, Domains, and Gene Structures of CaFAD Family Members in C. arabica

The conserved motifs, domains, and gene structures of *FAD* family proteins in *C. arabica* and *A. thaliana* were analyzed to elucidate their functional diversity and evolutionary relationships (Figure S1). However, no single conserved motif was shared across all 45 proteins. Members within the same subfamily exhibited a high structural similarity, indicating functional conservation. In the FAD and SLD subfamilies of both *C. arabica* and *A. thaliana*, motifs 3, 4, and 5 were consistently present, suggesting that these motifs may play a critical role in acyl-lipid desaturation. Conversely, the ADS subfamily contained only motifs 3 and 5. The DES subfamily was the most conserved, containing only motif 5, which highlights its specialized function in sphingolipid desaturation. The FAB subfamily contained motifs 1, 2, and 6, emphasizing its unique role in Δ-9 desaturation of stearoyl-ACP for monounsaturated fatty acid biosynthesis.

Domain analysis further supported the functional differentiation of the CaFAD subfamilies. The FAD subfamily exhibited four domains—PLN02505, membrane-FADs-like, PLN02498, and PLN02598—reflecting its functional complexity in fatty acid desaturation. The DES subfamily contained a single domain, PLN02579, consistent with its highly conserved role in sphingolipid Δ-4 desaturation. In contrast, the SLD subfamily displayed three domains—Cyt-b5, Delta6-FADS-like, and PLN03198—highlighting species-specific structural variations between *C. arabica* and *A. thaliana*. Interestingly, the ADS subfamily contained two domains—membrane-FADs-like and PLN02220—with partial domain overlap with the FAD subfamily, suggesting possible functional convergence. The FAB subfamily was structurally distinct, containing only the PLN00179 domain, which aligns with its simpler role in fatty acid synthesis.

Gene structure analysis revealed notable differences in the number of CDS (coding sequence) fragments across subfamilies, reflecting variations in gene complexity and regulation. Highly conserved genes, such as *CaFAD7*, *CaFAD6*, *CaFAD8*, *CaFAD9*, *CaFAD4*, and *CaSLD1*, contained a single *CDS* fragment, indicating stable structures optimized for specific enzymatic functions. In contrast, *CaFAD10* exhibited a more complex structure with 12 CDS fragments, suggesting potential alternative splicing or functional diversification. Genes within the same subfamily generally share similar structural patterns, reinforcing their evolutionary conservation and functional specialization.

The results highlight subfamily-specific motif and domain distributions, emphasizing structural diversity and evolutionary adaptation within the *CaFAD* family. The *FAD*, *SLD*, and *FAB* subfamilies exhibited greater motif and domain complexity, likely contributing to their broader enzymatic roles in lipid metabolism. Conversely, the *DES* subfamily, with fewer motifs and domains, showed greater specialization in sphingolipid desaturation.

### 2.4. Analysis of Chromosomal Distribution of CaFAD Genes in C. arabica

Chromosomal mapping of the *CaFAD* gene family was performed to investigate their genomic organization and distribution (Figure S2). A total of 21 *CaFAD* genes were mapped onto 12 chromosomes. Chromosome NC_039898.1 exhibited the highest diversity of *CaFAD* genes, carrying six members. The *CaFAB* genes were mainly located on chromosomes NC_039902.1, NC_039903.1, NC_039916.1, and NC_039917.1. The *CaFAD* genes were located on chromosomes NC_039898.1, NC_039899.1, NC_039900.1, NC_039908.1, and NC_039909.1. The *CaDSE* genes were located on chromosomes NC_039900.1 and NC_039901.1. The *CaADS* genes were located on chromosomes NC_039904.1 and NC_039905.1.

Interestingly, most of the *CaFAD* genes were positioned near the terminal regions of chromosomes, suggesting that these genes might have enhanced evolutionary adaptability and may be inherited more stably in *C. arabica*.

### 2.5. Analysis of Homologous Relationships Among CaFAD Family Members in C. arabica

**Figure 2:**
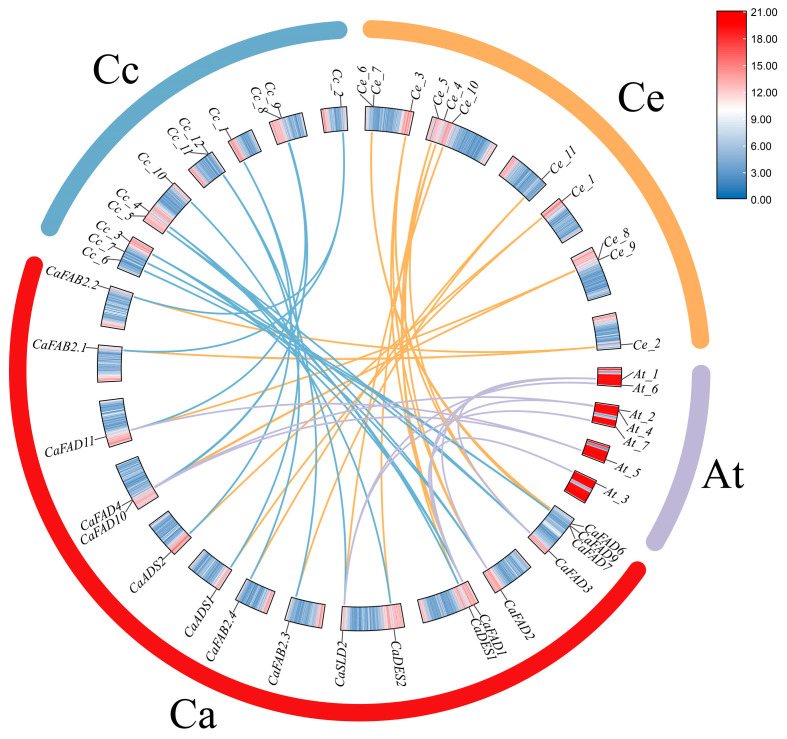
Homology analysis of *FAD* family members in *C. arabica*. Ca: *C. arabica*; Cc: *C. canephora*, Ce: *C. eugenioides*, and At: *A. thaliana*.

To investigate the evolutionary relationships and gene duplication events of the *FAD* gene family, a collinearity analysis was performed using *C. arabica* in comparison with *C. canephora*, *C. eugenioides*, and *A. thaliana* (Figure 2). The analysis identified 18 out of 21 *CaFAD* genes exhibiting collinearity with orthologous genes in the other three species, highlighting evolutionary conservation and gene duplication events across species. The collinearity results showed that the *CaFAD* genes had seven orthologous pairs distributed across four chromosomes in *A. thaliana*. In *C. canephora*, 12 orthologous genes were identified across six chromosomes, while *C. eugenioedes* shared 11 orthologous genes spanning six chromosomes with *C. arabica*. These patterns reflect the close evolutionary relationships between *C. arabica* and *C. canephora* and *C. eugenioedes*, which are its progenitor species, contributing to the formation of the allotetraploid genome of *C. arabica*.

Interestingly, one *CaFAD* gene was found to have collinear relationships with multiple genes in other species. Conversely, single genes in other species exhibited collinearity with multiple *CaFAD* genes in *C. arabica*. This suggests that gene duplication and divergence events have occurred extensively, contributing to the functional diversification of the *FAD* gene family.

### 2.6. Codon Usage Bias Analysis of the CaFAD Gene Family Members in C. arabica

Effective number of codons (ENC)-plot analysis was conducted to determine the extent to which natural selection influences codon usage patterns in the *CaFAD* gene family (Figure S3). The observed ENC values for the *CaFAD* genes were consistently below the standard expected curve, indicating a strong deviation from the neutral expectation. This deviation indicates that natural selection, rather than mutation pressure alone, is primarily responsible for shaping codon usage bias. Lower ENC values compared to expected values further imply that the codon usage preferences in *C. arabica* are subject to functional constraints related to gene expression optimization.

PR2-plot analysis was used to assess the base composition bias at the third codon position and the relative contributions of mutation pressure and selection (Figure S4). The majority of *CaFAD* genes clustered in the lower region of the plot, indicating a higher T bases frequency relative to A bases. This asymmetry suggests that codon usage bias is not random and is influenced by selective forces favoring specific base compositions. Additionally, the observed deviation of genes from the center point (0.5, 0.5) confirms that multiple factors, including natural selection, mutation pressure, and translational efficiency, contribute to the codon usage patterns in *C. arabica*.

A neutrality plot was generated to clarify how natural selection and mutation pressure each influence codon usage bias (Figure S5). The GC content of the first and second codon positions (GC12%) ranged from 43.04% to 48.57%, while the GC content at the third position (GC3%) ranged from 40.49% to 52.96%. The regression slope (0.109) and the low R^2^ value (0.0471) indicate a weak correlation between GC12% and GC3%, suggesting that natural selection exerts a stronger influence than mutation pressure. The lack of significant correlation (*p* > 0.05) reinforces the dominance of selective constraints in shaping codon usage patterns within the *CaFAD* genes.

### 2.7. Determination of Fatty Acid Variation Trends in C. arabica

**Figure 3:**
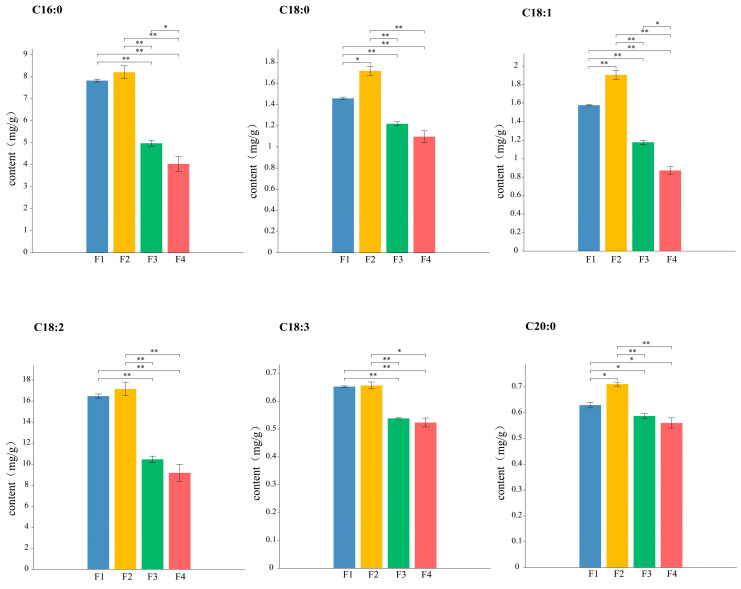
Determination of fatty acid contents in *C. arabica* seeds at different fruit development stages. F1, F2, F3, and F4 indicate 30, 80, 130, and 180 days post-flowering; * and ** indicate significant differences at *p* ≤ 0.05 and *p* ≤ 0.01.

To investigate the dynamic changes in fatty acids during seed development in *C. arabica*, gas chromatography was used to measure the fatty acid contents in *C. arabica* at 30, 80, 130, and 180 days post-flowering (Figure 3). The results demonstrated a general trend of higher fatty acid content in the early stages (F1 and F2) and gradual decreases in the later stages (F3 and F4). Among the measured fatty acids, linoleic acid was the most abundant component, followed by palmitic, stearic, and oleic acid. This indicates that polyunsaturated fatty acids dominate the lipid profile of mature *C. arabica* seeds. Among them, arachidic, oleic, and stearic acid showed the highest content at F2 and were significantly higher than the other stages, while they were decreased at F3 and were the lowest at F4. Linoleic acid, linolenic acid, and palmitic acid showed the same trend of highest at F2 and lowest at F4, but the differences in F1 and F2 were non-significant.

### 2.8. Expression Analysis of Genes Related to Fatty Acid Accumulation in C. arabica Seeds During Fruit Development

The expression profiles of fatty acid metabolism genes were analyzed at four developmental stages—30 (F1), 80 (F2), 130 (F3), and 180 (F4) days after flowering—of *C. arabica* using transcriptomic detection (Figure S6). Most *FAD* genes exhibited low or negligible expression throughout the developmental stages. Among the *CaFAD* gene family members, four genes displayed relatively high expression levels across the last three developmental stages (F2–F4). The *CaFAD4* gene showed a downward trend with the progress of the fruit development stage. However, the *CaFAD8* gene is highly expressed (F2–F4). The expression levels of the *CaFAB2.3* and *CaFAB2.4* genes showed the same trend, first decreasing with the lowest at F2 and then increasing, reaching the maximum in the F4 period.

### 2.9. Real-Time Fluorescence Quantitative Verification Analysis

**Figure 4:**
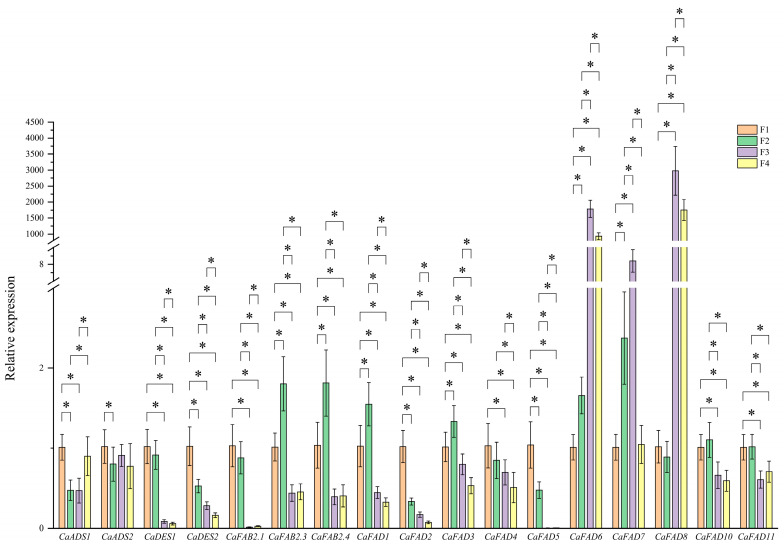
Validation by qRT-PCR. The expression levels of 17 *CaFAD* family genes during the four developmental stages—30 (F1), 80 (F2), 130 (F3), and 180 (F4) days post-flowering. The 2^−ΔΔCt^ data for the first developmental stage samples were used for normalizing the expression data. Results represent the mean of three biological replicates and two technical replicates (mean ± SD, *n* = 6). * indicates significant differences at *p* ≤ 0.05.

The expression profiles of *CaFAD* family genes during the four developmental stages—30 (F1), 80 (F2), 130 (F3), and 180 (F4) days post-flowering—were validated through qRT-PCR (Figure 4). The results demonstrated stage-specific expression patterns across the different gene subfamilies, suggesting the temporal regulation of the biosynthesis and desaturation processes of fatty acids. The expression of *CaADS1* was high during F1 and F4 but significantly lower in F2 and F3. In contrast, *CaADS2* exhibited its highest expression during F1, followed by a progressive decline across subsequent stages, reaching its lowest level at F4. This pattern indicates that *ADS* genes are active during the early stages of seed development and may contribute to initial fatty acid synthesis.

Both *CaDES1* and *CaDES2* displayed a continuous downward trend in expression levels from F1 to F4, suggesting their role is primarily limited to the early stages of lipid biosynthesis. This observation implies that *DES* genes may be involved in sphingolipid desaturation during the early phases of seed development. The *CaFAB2.1* gene showed high expression during F1 and F2, followed by extremely low levels in the later stages (F3 and F4), indicating a key role in early monounsaturated fatty acid biosynthesis. Conversely, *CaFAB2.3* and *CaFAB2.4* exhibited a biphasic trend, with low expression during F1, peak expression during F2 and F3, and a decline at F4, suggesting late-stage involvement in fatty acid modification and storage lipid accumulation.

The majority of *CaFAD* genes displayed high expression levels during the early and middle stages (F1–F3), followed by a reduction in F4, indicating their roles in early lipid biosynthesis and unsaturation processes. Notably, *CaFAD6* and *CaFAD8* showed opposite trends, with low expression in F1 and F2 and a sharp increase in F3 and F4, reaching their highest levels in the later stages. This suggests that *CaFAD6* and *CaFAD8* are key regulators of polyunsaturated fatty acid biosynthesis during seed maturation, potentially contributing to membrane fluidity and aroma compound precursors.

### 2.10. Subcellular Localization Analysis of the CaFAD8 Gene in C. arabica

**Figure 5:**
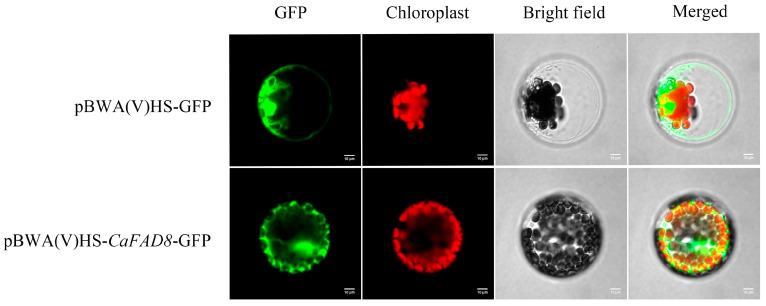
Analysis of subcellular localization of the *CaFAD8* gene in pBWA(V)HS-GFP (control vector) and pBWA(V)HS-*CaFAD8*-GFP (transient expression vector).

Transient expression assays in *A. thaliana* protoplasts were performed to investigate the subcellular localization of *CaFAD8* (Figure 5). In the experimental setup, *CaFAD8* was fused with GFP (green fluorescent protein) and expressed under a strong promoter. Fluorescence microscopy revealed that the *CaFAD8-GFP* signal was predominantly localized to the cell membrane and nucleus, as evidenced by the overlapping signals in GFP, chloroplast autofluorescence, and bright-field images.

Bioinformatic predictions using localization tools such as CELLO and WOLF PSORT further supported that *CaFAD8* is primarily localized to the cell membrane, aligning with its putative role in membrane-bound lipid metabolism. While the observed nuclear signal suggests potential secondary functions related to gene regulation or protein trafficking, the membrane localization underscores its primary functional role in fatty acid desaturation processes.

### 2.11. Fatty Acid Profiling in Seeds of CaFAD8-Overexpressing A. thaliana

**Figure 6:**
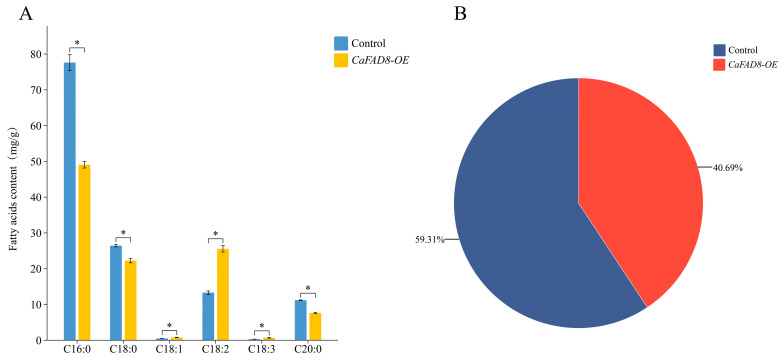
(**A**) Fatty acid content of six major fatty acids in CaFAD8-OE and wild-type *Arabidopsis*. (**B**) Relative proportion of total fatty acid in *CaFAD8-OE* and wild-type *Arabidopsis*. * indicates significant differences at *p* ≤ 0.05.

To explore the functional role of *CaFAD8* in fatty acid metabolism, the fatty acid contents of wild-type *A. thaliana* and *CaFAD8-OE A. thaliana* were measured (Figure 6A). It was found that the fatty acid content of the *CaFAD8-OE* transgenic plants was lower than that of the wild-type seeds. A comparative analysis of six major fatty acids highlighted significant changes in the composition between wild-type and transgenic plants (Figure 6A). Saturated fatty acids, i.e., stearic acid (C18:0), palmitic acid (C16:0), and arachidic acid (C20:0), were significantly reduced in *CaFAD8-OE* plants compared to wild-type (*p* ≤ 0.05). While unsaturated fatty acids were markedly elevated in transgenic plants (*p* ≤ 0.05). The pie chart represents the relative proportion of total fatty acids in wild-type and *CaFAD8-OE* plants, with *CaFAD8-OE* plants exhibiting higher total fatty acid content (59.31%) compared to wild-type plants (40.69%) (Figure 6B). Thus, the total fatty acid content in *CaFAD8-OE* was 18% less than the wild type. These findings demonstrate that *CaFAD8* enhances the degree of fatty acid desaturation, which is essential for membrane fluidity and aroma precursor biosynthesis in seeds.

## 3. Discussion

This study employed comprehensive bioinformatics analyses, including phylogenetic tree construction, conserved motif identification, domain characterization, and gene structure evaluation, to investigate the *CaFAD* gene family in *C. arabica*.

The phylogenetic tree of fatty acid desaturase (FAD) genes through homologous protein sequences from four species—*C. arabica*, *C. canephora*, *C. eugenioedes*, and *A. thaliana*—showed the five distinct subfamilies based on sequence homology and evolutionary distances. ADSs (acyl-CoA desaturases) are represented by members involved in the desaturation of acyl-CoA substrates, which are crucial for introducing double bonds into fatty acids. FAB (fatty acid biosynthesis) contains genes encoding stearoyl-ACP desaturases (*FAB2*) responsible for monounsaturated fatty acid synthesis in plastids [17]. SLDs (sphingolipid desaturases) include genes linked to the modification of sphingolipids, contributing to membrane fluidity and signaling pathways [19]. DESs (sphingolipid Δ-4 and Δ-8 desaturases) are related to the desaturation of sphingolipids essential for structural and functional integrity [20]. FADs (fatty acid desaturases) are the largest and most diverse subgroup, further divided into the following:

FAD2 converts oleic (C18:1) to linoleic acid (C18:2); FAD3/7/8 is involved in the synthesis of polyunsaturated fatty acids, including linolenic acid (C18:3); and FAD6 is associated with plastidial desaturation, contributing to membrane lipid composition.

The phylogenetic analysis revealed that members of the *CaFAD* gene family within the same subfamily displayed remarkably high sequence similarity, indicating a close genetic relationship [21]. The structural similarity and clustered distribution of some *CaFAD* genes on chromosomes suggest the formation of gene clusters, which may imply similar functions or coordinated expression patterns [22]. Gene duplication has been recognized as a key driver in gene family expansion and functional diversification [23]. Collinearity analysis with *C. canephora*, *C. eugenioides*, and *A. thaliana* demonstrated that multiple *FAD* genes in *C. arabica* exhibit collinear relationships with parental genes. Notably, one parental gene corresponded to two offspring genes, suggesting accelerated gene duplication events that could result in gene redundancy or novel functions [24].

Codon usage bias analysis revealed that the *CaFAD* gene family in *C. arabica* is influenced predominantly by natural selection and mutation pressure, with natural selection playing a more prominent role. This highlights the possibility that evolutionary pressures, such as natural selection, may also drive gene duplication events [25].

Analysis of fatty acid composition in *C. arabica* seeds across four developmental stages identified linoleic acid, palmitic acid, oleic acid, and stearic acid as major components. Oleic acid, a precursor to linoleic acid, plays a pivotal role in determining total fatty acid content. Measurements of fatty acid levels during the seed development stages revealed an initial increase followed by a gradual decrease, with the most significant decline occurring between the F2 and F3 stages. The relative stability between the F3 and F4 stages suggests metabolic transitions during maturation. At maturity, the lowest fatty acid content was observed, likely due to conversion into other organic compounds during maturation [26]. Variations in fatty acid trends across crops further emphasize species-specific differences.

Previous studies have identified more than 10 FAD members in plants such as flax [27], *Juglans regia* L. [6], and tomato [28], with FAD2 being a primary focus. The *FAD2* gene regulates the conversion of oleic acid to linoleic acid, a critical step in unsaturated fatty acid biosynthesis [29]. Phylogenetic and structural analyses in this study indicated that the *CaFAD8* gene shares high homology with the *AtFAD2* gene. Expression pattern analysis revealed significant variation during seed development, with sustained high expression levels in later stages. This suggests ongoing fatty acid synthesis even during maturation. Interestingly, while fatty acid levels decreased, *CaFAD8* expression remained elevated—a phenomenon also observed in *Juglans regia* [6] and *Carthamus tinctorius* [30].

Subcellular localization experiments confirmed that *CaFAD8* resides on the plasma membrane, aligning with the localization patterns of *FAD2* and *FAD3/7/8* genes [14,31,32]. The heterologous expression of *CaFAD8* in *A. thaliana* led to increased levels of oleic, linoleic, and linolenic acids despite a reduction in total fatty acid content. This suggests that *CaFAD8* enhances fatty acid unsaturation by facilitating oleic acid conversion to linoleic acid. Consistent findings across various studies underscore the role of exogenous *FAD* gene expression in promoting unsaturation rather than increasing total fatty acid content [33,34,35,36,37].

Furthermore, in higher plants, the *FAB2* gene is expressed in various organs, with peak expression in developing seeds. This study observed a positive correlation between oleic acid content and the expression of *CaFAB2.3* and *CaFAB2.4* genes in *C. arabica* seeds. Both genes are localized to chloroplasts, suggesting their involvement in oleic acid biosynthesis during seed development. Studies have also demonstrated the role of *FAB2* genes in abiotic stress responses [38]. For example, *Carthamus tinctorius* exhibited increased *CtFAD* expression under temperature and salt stress, resulting in higher C18:3 levels [39]. Similarly, the stress-induced expression of *SAD* genes was observed in *Camellia sinensis* and *Linum usitatissimum* [40,41].

Numerous studies have clearly demonstrated that, throughout the coffee cultivation process, the fatty acid composition within coffee is acutely sensitive to multiple factors. These include altitude variations [42], average temperature fluctuations [43], and the inherent genetic traits of the coffee plants [44]. However, conspicuously, these studies have failed to comprehensively consider the profound influence that relevant genes exert on the synthesis of coffee fatty acids. Collectively, these findings suggest that *CaFAB2* and *CaFAD* genes not only regulate oleic acid biosynthesis during *C. arabica* seed development but may also enhance resistance to abiotic stresses, underscoring their potential roles in improving crop resilience and quality. When it comes to the roasting of coffee seeds, it is remarkable that, despite unsaturated fatty acids constituting merely 1% of the total coffee lipids, they possess high reactivity during roasting [45]. This reactivity readily triggers the generation of a substantial quantity of volatile compounds. These volatiles not only play a pivotal role in shaping the flavor of coffee beverages but also wield a profound influence on the professional evaluation and in-depth analysis of coffee. Consequently, this research is dedicated to a meticulous exploration and clarification of the molecular mechanisms underlying the formation of fatty acids in *C. arabica* seeds. It is anticipated that the findings will furnish valuable theoretical insights and practical guidance for the subsequent breeding of Arabica coffee varieties characterized by high oil content and uniquely novel flavors.

## 4. Materials and Methods

### 4.1. Identification and Structural Analysis of CaFAD Gene Family Members in C. arabica

Reference genome sequences for *Coffea arabica* L., *C. canephora* Pierre ex A. Froehner, and *C. eugenioides* S. Moore (downloaded from the NCBI database https://www.ncbi.nlm.nih.gov/, accessed on 1 November 2023) were used to construct local databases for subsequent analyses (Table S2). Protein sequences of 24 *Arabidopsis FAD* genes were obtained and used as queries for local BLASTP searches against the coffee protein sequences with an E-value threshold of <1 × 10^−10^. Redundant sequences were removed, and the candidate *CaFAD* gene family member sequences were identified (Table S2).

The Expasy ProtParam tool (https://www.expasy.org/, accessed on 5 November 2023) was used to analyze the properties of the *CaFAD* gene family members, i.e., molecular weight, number of amino acids, aliphatic index, isoelectric point (pI), instability coefficient, and grand average of hydropathicity (GRAVY).

For phylogenetic analysis, *FAD* genes of *C. arabica*, *C. canephora*, *C. eugenioides*, and *A. thaliana* were aligned using the MUSCLE algorithm in MEGA7 (Table S3). The neighbor-joining (NJ) method was adopted to construct a phylogenetic tree, and Chiplot (https://www.chiplot.online/, accessed on 11 November 2023) was used to enhance visualization. Conserved motifs and functional domains were analyzed using MEME (http://meme-suite.org/, accessed on 11 November 2023) and the NCBI Batch CD-Search Tool (https://www.ncbi.nlm.nih.gov/Structure/bwrpsb/bwrpsb.cgi, accessed on 11 November 2023). Results were visualized using TBtools v2.016 software.

### 4.2. Genetic Evolution Analysis of CaFAD Gene Family

The TBtools v2.016 software was used to perform chromosomal localization of the *CaFAD* genes [46]. Collinearity analyses were conducted among the genomes of *C*. *arabica*, *C. canephora*, *C. eugenioides*, and *A. thaliana*.

The effective number of codons (ENC) was estimated through the Bioinformatics Cloud online tool (http://112.86.217.82:9929/#/, accessed on 21 October 2024). ENC-plot analysis was carried out using the expected value to determine the codon usage bias, taking base mutations as the standard curve. The calculation method for the standard curve, as described by Wang et al. [47], was used.

$$ENC=2+GC3+\frac{29}{{GC3}^{2}+(1-{GC3)}^{2}}$$

The proportions of the nucleotide bases A, T, G, and C at the third codon position were determined using MEGA7 and labeled as A3, T3, G3, and C3, respectively. Codon usage bias was analyzed by plotting G3/(G3 + C3) on the horizontal axis and A3/(A3 + T3) on the vertical axis. Additionally, the GC content at the first, second, and third codon positions was assessed using the Bioinformatics Cloud online platform (http://112.86.217.82:9929/#/, accessed on 21 October 2024), with the results denoted as GC1, GC2, and GC3. The average of GC1 and GC2, referred to as GC12, was also calculated. Neutrality plots were created using GC3 as the x-axis and GC12 as the y-axis.

### 4.3. Determination of Fatty Acid Variation in C. arabica

The fatty acid content in *C. arabica* seeds was determined across four fruit developmental stages: 30, 80, 130, and 180 days after flowering—corresponding to BBCH 71 (fruit set, 10% size), BBCH 75 (50% size), BBCH 77 (70% size), and BBCH 80 (physiological maturity) (Figure S7). Seeds after collection were immediately ground in liquid nitrogen, freeze-dried, and sieved (80-mesh) for uniformity [48]. A sample of 0.1 g was extracted using 2 mL of petroleum/benzene mixture (1:1, *v*/*v*). Methylation was catalyzed by adding 2 mL of KOH (0.4 mol/L) in methanol and incubated in a water bath at 80 °C for 15 min. Then, 4 mL of saturated sodium chloride solution was added to facilitate phase separation. The mixture was shaken thoroughly and allowed to stand for stratification. The supernatant containing fatty acid methyl esters (FAMEs) was carefully collected and filtered through a 0.22 µm organic filter membrane.

FAMEs were analyzed using a GC-MS with a DB-5 column (60 m × 250 μm × 0.25 μm). High-purity helium (99.99%) was used as a carrier gas at a flow rate of 1.3 mL/min. 1 µL of filtered supernatant was injected in the splitless mode. The temperature program ranged from 60 °C to 280 °C, while the ion source and quadrupole temperatures were set to 230 °C and 150 °C, respectively. All experiments were performed with three biological replicates to ensure reproducibility. Fatty acids were identified and quantified by their retention times and mass spectra against those of known standards.

### 4.4. Gene Expression Analysis of CaFAD Genes in C. arabica at Different Stages

RNA was extracted from *C. arabica* seeds at the four developmental stages, and RNA-seq sequencing was conducted using Illumina HiSeq. Transcriptome data have been submitted to the BIG Submission, BIG Sub (accession number PRJCA034229). *FAD* gene expression was analyzed using the method of the fragments per kilobase of exon model per million mapped fragments (FPKM). Genes with average FPKM values > 1 were selected for further analysis (Table S4). The expression heatmap was drawn using TBtools v2.016.

For qRT-PCR validation, primers were designed using the Primer-BLAST website (https://www.ncbi.nlm.nih.gov/tools/primer-blast/, accessed on 10 December 2023) (Table S5). Relative expression levels were normalized with 24S rRNA [49] and calculated using the 2^−ΔΔCt^ method. The qPCR reaction (20 µL) included 0.8 µL primers, 6 µL of ddH2O, 10 µL of TB Green Premix Ex Taq II, 2 µL of cDNA, and 0.4 µL of ROX Dye II (50×). Thermal cycling was conducted for a total of 40 cycles, beginning with a pre-denaturation step at 95 °C for 30 s, followed by denaturation at 95 °C for 5 s and a final step of annealing at 60 °C for 34 s.

### 4.5. Subcellular Localization Analysis of CaFAD8

The full-length *CaFAD8* sequence was amplified with restriction sites (KponI and XbaI), and cloning was performed in the pBWA(V)HS-GFP vector. Recombinant plasmids were transferred into *Escherichia coli* DH5α cells for cultivation. Positive bacterial cultures were selected, and plasmids were extracted for identification and sequencing. The leaves of *A. thaliana* (25–30 days old) were cut into 1 mm pieces and enzymatically hydrolyzed at 24 °C for 4 h. After filtration through a 40 μm filter mesh and centrifugation at 300 rpm for 3 min, the supernatant was removed. Subsequently, they were washed twice with 10 mL of pre-cooled W5 solution at 300 rpm for 3 min. After being suspended in 500 μL of MMG solution, microscopic examination was performed. The control vector (pBWA(V)HS-GFP) and the transient expression vector (pBWA(V)HS-*CaFAD8*-GFP) were transfected into protoplasts using the PEG4000-mediated method and allowed to stand at room temperature for 30 min. The reaction was terminated by dilution with 1 mL of W5. Protoplasts were collected by centrifugation at 300 rpm for 3 min, and the supernatant was removed. They were washed 1–2 times with 1 mL of W5. Finally, 1 mL of W5 solution was added, and they were subjected to dark culture at 28 °C for 18–24 h. Finally, the supernatant was removed, leaving only approximately 100 μL of protoplasts for laser confocal microscopy observation. The main instruments used in this experiment include the fully automatic gel imaging analysis system (ZF-258), constant voltage and current electrophoresis apparatus (DYY-8D), digital display constant temperature water bath (HH-S3), centrifuge (Thermo Sorvall ST16R), and laser confocal microscope (Nikon C2-ER), etc. Green fluorescent protein (GFP): excitation light 488 nm, emission light 510 nm. Chloroplast excitation light 640 nm, emission light 675 nm.

### 4.6. Functional Analysis of CaFAD8 in Transgenic Arabidopsis thaliana

To construct the *CaFAD8* overexpression vector, the *CaFAD8* CDS after PCR amplification was cloned into the *pBWA(V)HS* vector containing the 35S promoter. After transformation into *Agrobacterium tumefaciens* GV3101, Arabidopsis plants were infected via floral dip. Seeds from transformed plants were harvested and screened until *CaFAD8-OE* homozygous lines were obtained. For fatty acid analysis, seeds from transgenic plants were analyzed for fatty acid content using the method described in Section 2.3.

## 5. Conclusions

This study comprehensively analyzed the *CaFAD* gene family in *Coffea arabica*, revealing gene duplication events likely driven by natural selection and mutation pressure, which contributed to its evolutionary expansion and functional diversity. Expression profiling and fatty acid content analysis demonstrated that fatty acids were abundant in the early stages of seed development but declined during maturation. Among the identified genes, *CaFAB2.3*, *CaFAB2.4*, *CaFAD4*, and *CaFAD8* exhibited significant expression changes, with *CaFAD8* showing the most distinct trend, correlating with unsaturated fatty acid synthesis. Subcellular localization confirmed *CaFAD8*’s association with the cell membrane, supporting its role in membrane-bound lipid metabolism. Heterologous expression in *A. thaliana* validated its function in enhancing unsaturation, increasing oleic acid, linoleic acid, and linolenic acid while reducing total fatty acid content. These findings identify *CaFAD8* as a key regulator of unsaturated fatty acid biosynthesis in *C. arabica*, providing insights into its molecular mechanisms and laying the groundwork for breeding high-oil-content coffee varieties with improved aroma and flavor profiles.

## Data Availability

Data are contained within the article and Supplementary Materials.

## Supplementary Materials

The following supporting information can be downloaded at https://www.mdpi.com/article/10.3390/ijms26031023/s1.
