# Supplementary figures and images for: Comprehensive Analysis of CaFAD Genes Involved in Fatty Acid Accumulation in Coffea arabica and Functional Characterization of CaFAD8 in Transgenic Arabidopsis thaliana
Source: Int J Mol Sci. 2025 Jan 25;26(3):1023. doi: 10.3390/ijms26031023 (PMC11816918; doi:10.3390/ijms26031023)

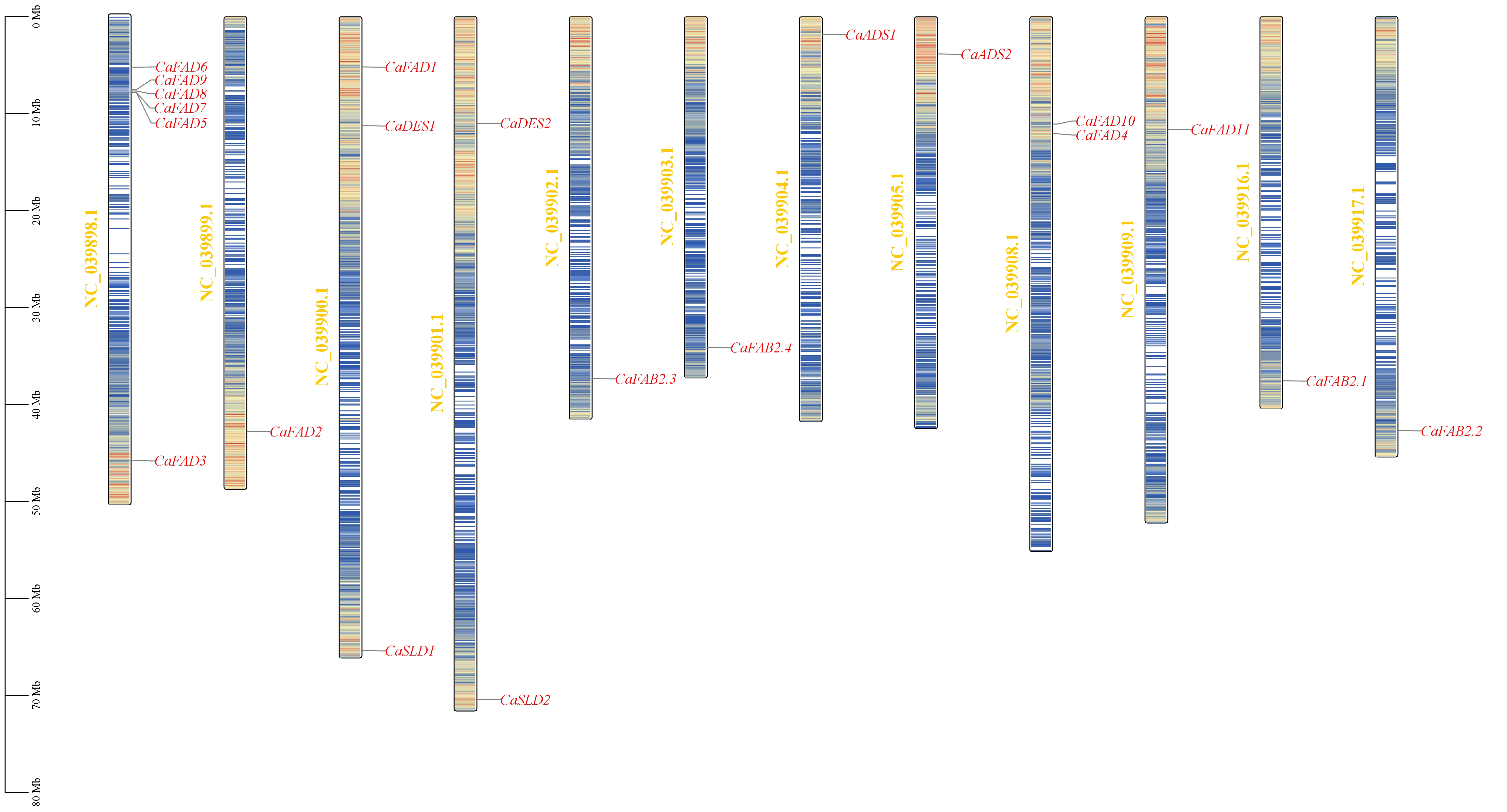

Supplement: Supplementary file 1 [file ijms-26-01023-s001.zip › Supplementary material/Figure S2. Distribution of FAD gene family members in C. arabica across the chromosomes.jpg]

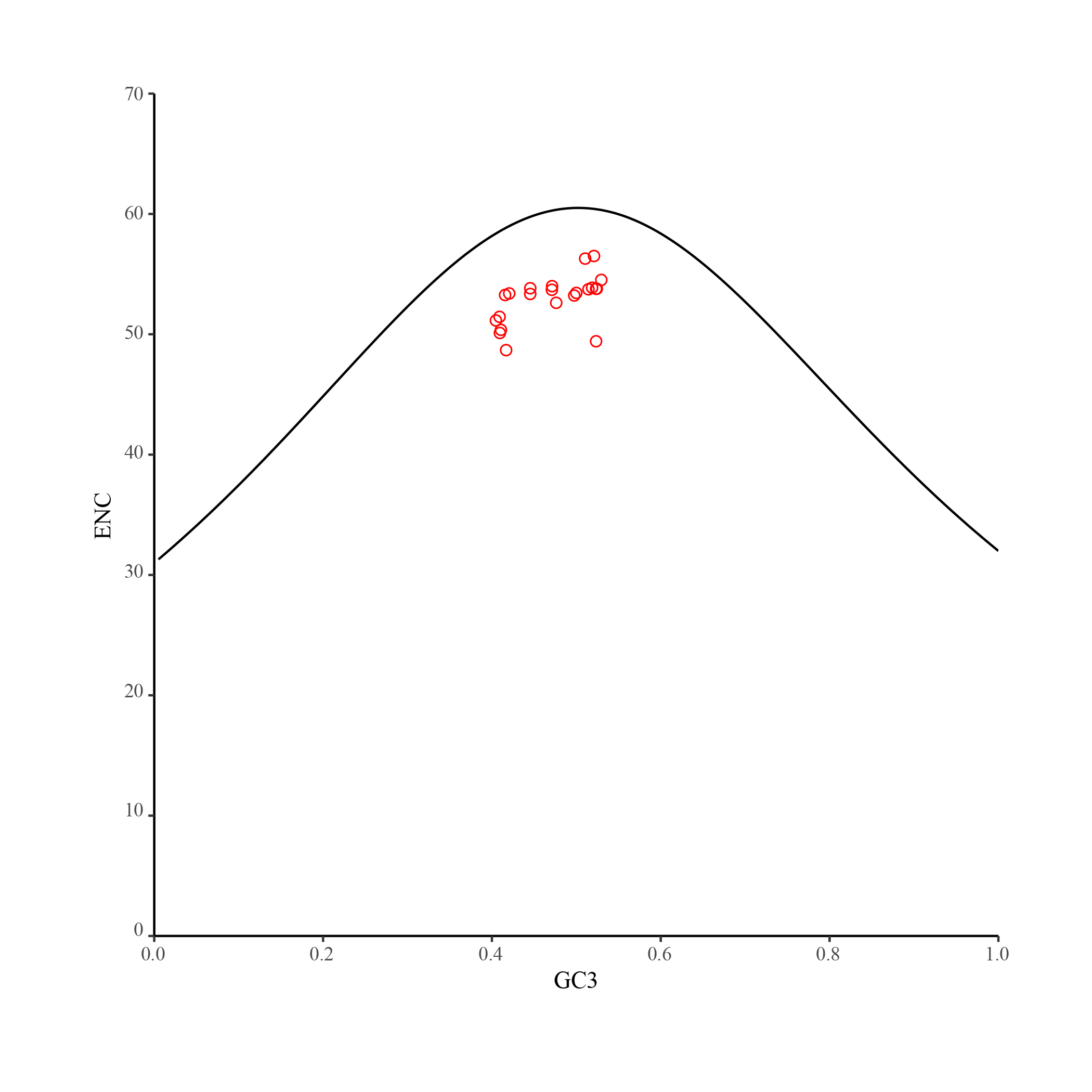

Supplement: Supplementary file 1 [file ijms-26-01023-s001.zip › Supplementary material/Figure S3. ENC plot analysis.jpg]

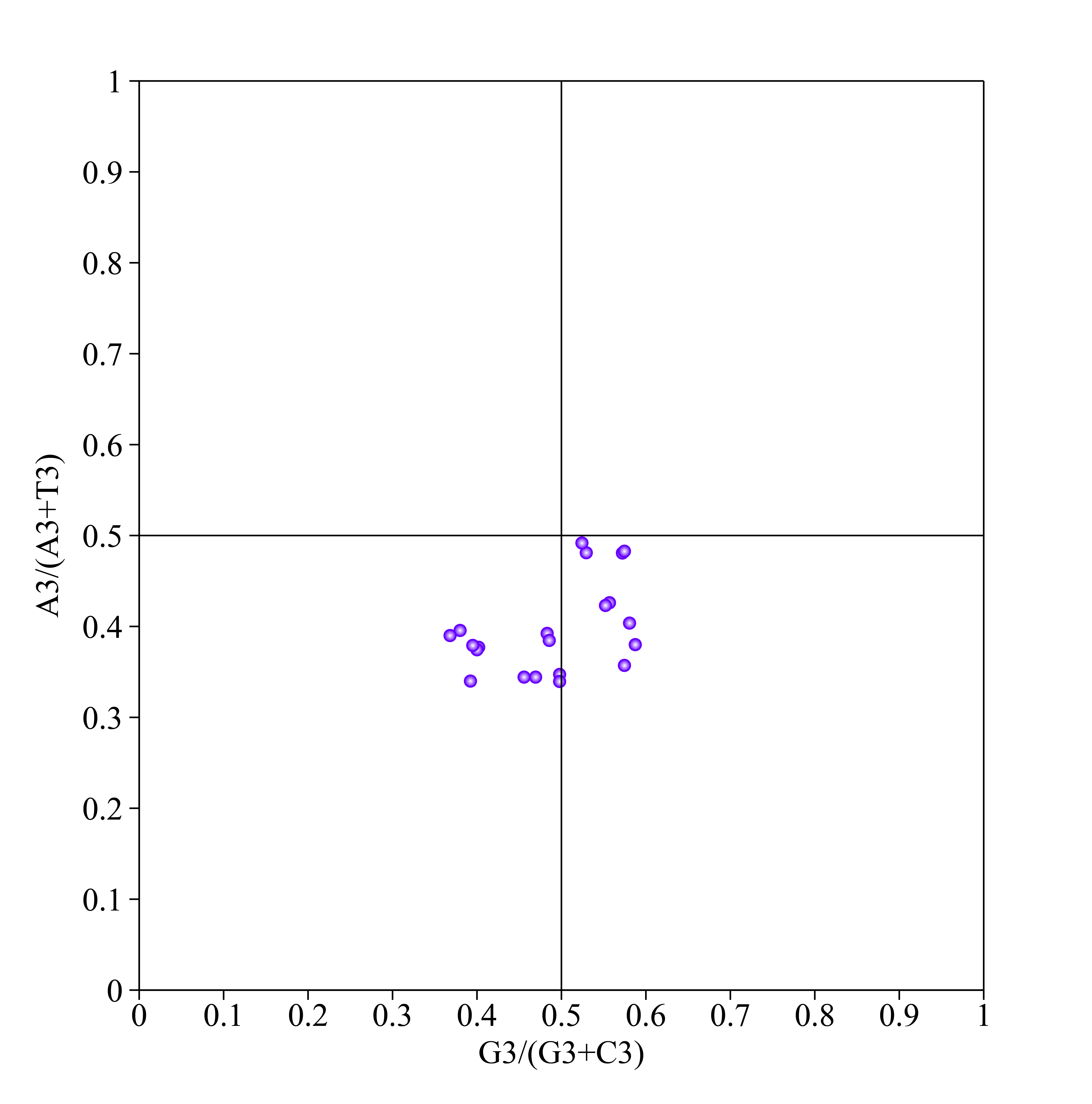

Supplement: Supplementary file 1 [file ijms-26-01023-s001.zip › Supplementary material/Figure S4. PR2-plot analysis.jpg]

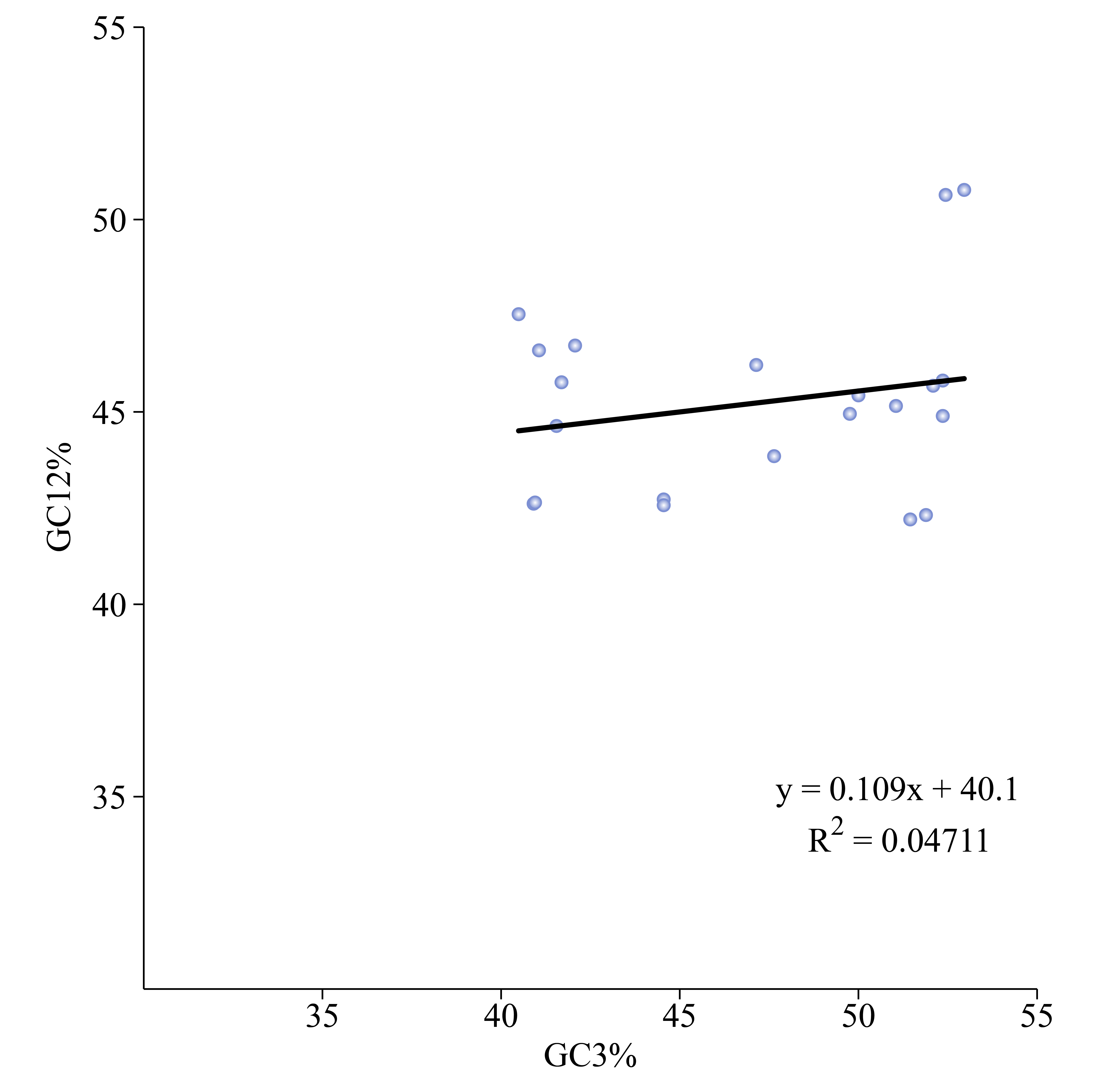

Supplement: Supplementary file 1 [file ijms-26-01023-s001.zip › Supplementary material/Figure S5. Neutral plot analysis.jpg]

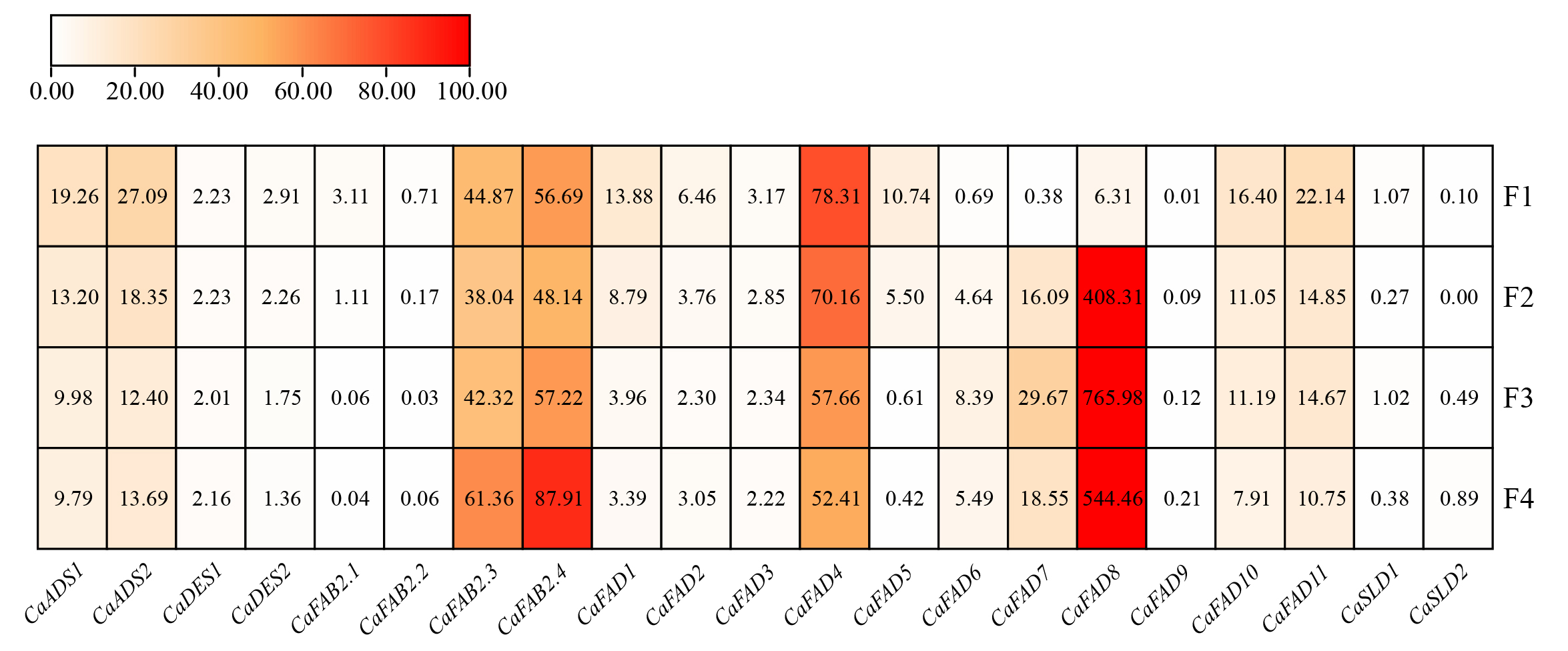

Supplement: Supplementary file 1 [file ijms-26-01023-s001.zip › Supplementary material/Figure S6. Expression analysis of genes related to fatty acid accumulation in C. arabica seeds at different fruit development stages..jpg]
